# Documentation of comorbidities, lifestyle factors, and asthma management during primary care scheduled asthma contacts

**DOI:** 10.1038/s41533-024-00360-3

**Published:** 2024-03-09

**Authors:** Jaana Takala, Iida Vähätalo, Leena E. Tuomisto, Onni Niemelä, Pinja Ilmarinen, Hannu Kankaanranta

**Affiliations:** 1 Department of Respiratory Medicine, Seinäjoki Central Hospital, Wellbeing Services County of South Ostrobothnia, Seinäjoki, Finland; 2 Seinäjoki Health Care Centre, Wellbeing Services County of South Ostrobothnia, Seinäjoki, Finland; 3 Tampere University Respiratory Research Group, Faculty of Medicine and Health Technology, Tampere University, Tampere, Finland; 4 Department of Laboratory Medicine, Seinäjoki Central Hospital, Wellbeing Services County of South Ostrobothnia, Seinäjoki, Finland; 5 Tampere University, Tampere, Finland; 6 Krefting Research Center, Department of Internal Medicine and Clinical Nutrition, Institute of Medicine, Sahlgrenska Academy, University of Gothenburg, Gothenburg, Sweden

**Keywords:** Asthma, Clinical trial design, Respiratory tract diseases, Public health, Health services

## Abstract

Systematically assessing asthma during follow-up contacts is important to accomplish comprehensive treatment. No previous long-term studies exist on how comorbidities, lifestyle factors, and asthma management details are documented in scheduled asthma contacts in primary health care (PHC). We showed comorbidities and lifestyle factors were poorly documented in PHC in this real-life, 12-year, follow-up study. Documented information on rhinitis was found in 8.9% and BMI, overweight, or obesity in ≤1.5% of the 542 scheduled asthma contacts. Of the 145 patients with scheduled asthma contacts, 6.9% had undergone revision of their inhalation technique; 16.6% had documentation of their asthma action plan. Screening of respiratory symptoms was recorded in 79% but nasal symptoms in only 15.5% of contacts. Lifestyle guidance interventions were found in <1% of contacts. These results, based on documented patient data, indicate a need exists to further improve the assessment and guidance of asthma patients in PHC.

## Introduction

Asthma is a long-term variable respiratory disease^1^ with low remission rates if diagnosed at adult age^2–4^. The reasons for poor asthma control are complex and may include patient-, healthcare-, and therapy-related issues^5^. Comorbidities such as obesity, allergy, rhinitis, gastroesophageal reflux, psychiatric disorders, obstructive sleep apnea, bronchiectasis, and sensitivity to non-steroidal anti-inflammatory drugs (NSAIDs) are common in asthmatics^6–13^. Asthma-related comorbidities may contribute to poor disease control by aggravating or mimicking symptoms, thus making it more difficult to distinguish true, severe asthma from difficult-to-treat asthma^7,9^. This, in turn, may lead to overtreatment or undertreatment with anti-asthma medication or lead to misdiagnosis^6,7^. The risk of multiple, non-respiratory comorbidities has been shown to be higher in late-onset asthma^11,14^. Socioeconomic factors^15,16^, poor adherence to inhaled corticosteroids (ICS)^1,5^, problems in inhalation technique^1,5^ and lifestyle factors such as smoking^17^ and low physical activity^18^, are also, in addition to comorbidities, associated with poorer asthma control. Self-management, including education, personal action plan, and structured follow-up, are strongly recommended as key components of asthma care and are shown to improve asthma control and reduce patients’ use of health-care resources and costs^19,20^.

The aforementioned aspects underscore why regular holistic assessment and guidance of asthma patients is important^1^. Annual follow-up reviews do not, unfortunately, occur often according to guidelines^21–24^, even in severe asthma^25,26^, that is shown to be underdiagnosed in primary health care (PHC)^27^. The lack of regular follow-up is not limited only to primary care^21,22^, when studies with patients from both primary and specialised care have also suggested that ~50–70% of patients^23,24^ and over 30% in severe asthma^26^ had no annual planned contacts. Moreover, adherence to asthma guidelines has been reported to be suboptimal among health-care practitioners^21,28–30^. Based on those factors, one might assume asthma evaluation is largely carried out, e.g., during visits made for other conditions or for acute exacerbations. However, in visits where asthma is not the only issue of attention, or if the visit has been made, e.g., due to acute exacerbation, no similar possibility for a comprehensive asthma assessment exists, arguably, except in planned follow-up contacts. Thus, it can be considered important to discover how systematically asthma assessments are performed in visits that focus purely on asthma to evaluate how guidelines are implemented in asthma monitoring.

The main responsibility for asthma treatment was shifted to PHC within the Finnish National Asthma Programme^31^. Important programme goals, were, e.g., active asthma treatment, use of lung function tests as part of control assessment, patient education together with guided self-management, and possible trigger evaluation^31^. Our previous long-term study showed that adherence to lung function measurements, especially to spirometry, in assessing asthma control was high in PHC^22^. Conversely, the frequency of asthma follow-up contacts was insufficient^22^, as was smoking data and smoking cessation documentation^32^. Previous studies, mainly based on self-reports or short-term follow-ups, have suggested several shortcomings in asthma management in PHC, including asthma control assessment^30,33,34^, self-care guidance^33,34^, rhinitis screening and treatment^35,36^, inhaler technique review^30,34^ and physical activity, nutrition and alcohol consumption assessment^34^.

To the best of our knowledge, no previous long-term real-life studies exist on how comorbidities, lifestyle factors, and asthma management details, such as inhalation technique and medication data, are screened and documented in scheduled asthma contacts during long-term follow-up in PHC, being the current study’s aim. Our additional aim was to assess whether there are differences according to who encountered the patient at the follow-up visit (GP, nurse, or both).

## Methods

### Setting of the SAAS study

The study was part of the Seinäjoki Adult Asthma Study (SAAS), a real-life, single-centre, 12-year follow-up study of 203 patients with new-onset asthma diagnosed at adult age (≥15 years). The details of the SAAS study protocol with inclusion, exclusion and specific diagnostic criteria were published separately (eTable 1)^37^. The original study cohort comprised 256 patients with new-onset asthma diagnosed between 1999 and 2002 in Seinäjoki Central Hospital’s respiratory department by a respiratory physician based on typical symptoms and was confirmed by objective lung function measurements. Smokers and patients with concomitant COPD or other comorbidities were also included^37^. The patients were treated and monitored by their personal physicians after the diagnosis was confirmed and the medication started, mostly in PHC, according to the Finnish National Asthma Programme^31^ as described previously^22,37^. The patients were invited to follow-up visit in the respiratory department after 12 years (mean 12.2, range 10.8–13.9 years). Of the original study population, 53 patients were lost to follow-up (Supplementary Figure 1) and 203 patients completed a follow-up visit, where asthma status, disease control, comorbidities, and medication were evaluated using structured questionnaires and lung function was measured^37,38^. The participants in the follow-up visit gave written informed consent to the study protocol approved by the Ethics Committee of Tampere University Hospital, Tampere, Finland^37^. All data of the asthma-related health-care contacts (*n* = 3639) during the 12-year period were collected from PHC, occupational health care, private clinics, and hospitals in addition to the data gathered at diagnostic and follow-up visits, as previously described^22,37^. Each patient, on average, had 4 [interquartile range (IQR) 1–6] scheduled asthma contacts and, overall, 15 (IQR 9–23) asthma-related health-care contacts during the follow-up period. The SAAS study flowchart and schematic presentation are shown in the supplementary material (eFig. 1; eFig. 2). The SAAS study is registered at www.ClinicalTrials.gov with identifier number NCT02733016^37^.

### Study design and population

**Fig. 1:**
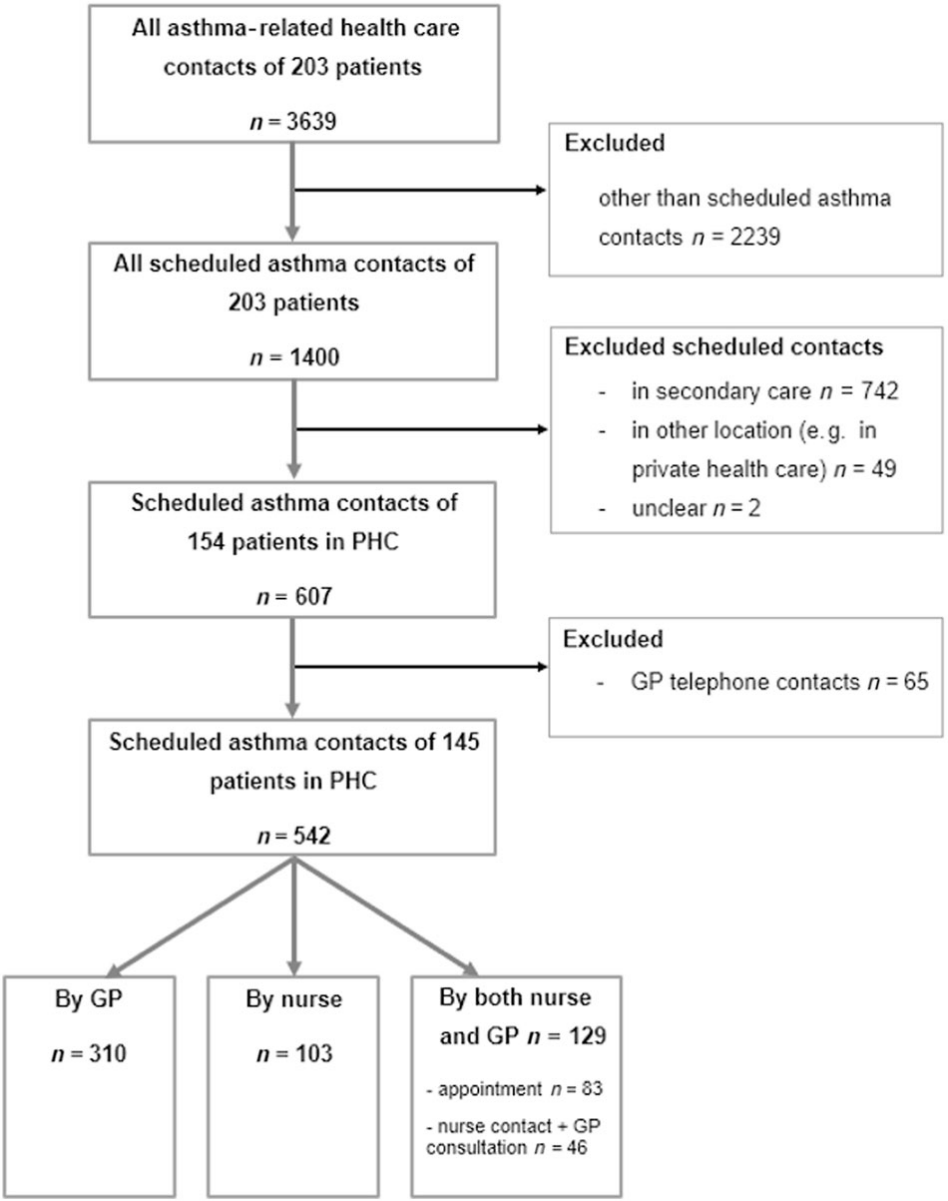
The flowchart of the study.

All asthma-related health-care contacts (*n* = 3639) of the 203 patients during the 12-year follow-up period were retrospectively assessed in the present study (Fig. 1). The following definitions were used to categorise different asthma contact types:

- *Primary health care (PHC) contact*: contact made in primary health care centre or in occupational health care.
- *Secondary care contact*: contact in specialised care in respiratory department.
- *Private health care contact*: contact in private health care.
- *Doctor/GP contact*: contact with only GP participating in the asthma assessment.
- *Nurse contact*: contact with only nurse participating in the asthma assessment.
- *Both doctor/GP and nurse contact*: contact with both professionals participating in the asthma assessment.
- *Scheduled asthma contact*: planned monitoring contact that purely focused on asthma.
- *Office-based contact*: patient encountered the professional face-to-face.
- *GP telephone contact*: a doctor phone call to a patient regarding asthma.
- *Other than scheduled asthma contact*: includes other asthma-related health-care contacts, excluding planned asthma contacts.
- *Unclear type of contact*: the exact type for the contact could not be determined.
- *All asthma-related health-care contacts*: includes scheduled asthma contacts and contacts made for infection, exacerbation, or for asthma and other reason.

We excluded contacts made for infection, exacerbation or for asthma and other reason of all the asthma-related contacts (*n* = 3639) (Fig. 1). Of the total 203 patients, 154 had scheduled asthma contacts (*n* = 607) in PHC, while 20 patients’ follow-up was arranged in secondary care or in private health care, and 29 patients had no planned follow-up contacts between the diagnostic visit and the 12-year follow-up visit as our previous study described^22^. Overall, of all scheduled asthma contacts during the 12-year follow-up, 742 occurred in secondary care, 49 in other locations, and two contacts’ locations were unclear. Our previous studies described the occurrence of scheduled asthma contacts in PHC and the overall occurrence of asthma follow-up contacts in the SAAS study population^22,23^.

We included all office-based, scheduled asthma contacts (*n* = 542) in PHC in this study. GP telephone contacts (*n* = 65) were excluded because these were often short phone calls, e.g., due to a previously made medication change, discussion of current test results, or the need for specific medical certificates, and were basically not intended to replace a more comprehensive face-to-face assessment. Nine patients had only telephone contacts of the total population of 154 patients with scheduled asthma contacts in PHC. Thus, after exclusion of GP telephone contacts, the total population in this study with office-based, scheduled asthma contacts was 145 (Fig. 1). The data of 145 patients and the data gathered from their office-based, scheduled asthma contacts in PHC were manually collected and evaluated.

### Evaluation of the content of asthma contacts according to professional

We divided the 542 scheduled contacts into three groups (GP, nurse, or both) to evaluate whether differences exist in how comorbidities and other asthma-related data were documented. The contacts were evaluated according to who was responsible for the patient in the office-based asthma follow-up contact: 310 were GP contacts,103 were nurse contacts, and 129 were combined GP and nurse contacts (Fig. 1). Of 129 combined GP and nurse contacts, the patient first met the nurse and the GP thereafter at the same visit in 83 contacts; the patient met the nurse and the GP was only consulted in 46 contacts.

### Collection of data on comorbidities, lifestyles, symptoms, and asthma management

We collected information on the comorbidities associated with asthma, including obesity, nasal conditions, gastroesophageal reflux, obstructive sleep apnea (OSA) and intolerance to NSAIDs. The evaluated nasal conditions included chronic/allergic rhinitis, sinus infections and nasal polyps. We also collected information on documentation of obesity-related lifestyle factors (including exercise habits, diet, and alcohol use), asthma symptoms, and patient guidance. All documented medication and data considering inhalation technique were manually collected from patient records and evaluated. Our previous studies described performance of lung function tests, documentation of smoking data and smoking cessation activities in scheduled asthma contacts in PHC^22,32^.

### Assessment of lung function, asthma control, severity, and other clinical parameters

The lung function measurements were performed with a spirometer according to international recommendations at the diagnostic visit and at the 12-year follow-up visit^37,38^. The fraction of exhaled nitric oxide (FeNO) was measured with a portable rapid-response chemiluminescent analyser according to American Thoracic Society standards^39^ (flow rate 50 mL·s^−1^; NIOX System, Aerocrine, Solna, Sweden). Venous blood was collected, and white blood cell differential counts were determined. Total immunoglobulin (Ig)E levels were measured by using ImmunoCAP (Thermo Scientific, Waltham, USA). Laboratory assays were performed in an accredited laboratory (SFS-EN ISO15189:2013) of Seinäjoki Central Hospital.

Patients completed the Asthma Control Test (ACT) and Airways Questionnaire 20 (AQ20) in the 12-year follow-up visit^40,41^. An asthma control assessment was performed according to the Global Initiative for Asthma (GINA) 2010 report^42^. Severe asthma assessment was performed according to the ERS/ATS severe asthma guideline 2014^43^. Adherence to inhaled corticosteroid (ICS) medication was evaluated by comparing the dispensed doses to the prescribed doses for the whole 12-year period as our previous studies described^44,45^. The prescribed dose in each patient was calculated based on medical records, and the dispensed ICS, short-acting β_2_-agonist (SABA) and oral corticosteroids were obtained from the Finnish Social Insurance Institution, which records all purchased medication from all Finnish pharmacies^44,45^. The 12-year adherence and annual adherence for each patient were calculated using specific formulas previously described, considering aspects from the medication possession ratio (MPR) and proportion of days covered (PDC)^44^.

Information on alcohol consumption was assessed by detailed structured questionnaires at the 12-year follow-up visit. Heavy alcohol consumption was evaluated by self-report, GT-CDT index or both. An alcohol consumption assessment was performed according to the US definitions for alcohol consumption by portions/week (portion indicates 14 g alcohol)^46^. Serum levels for carbohydrate-deficient transferrin (CDT) were measured by a turbidimetric immunoassay (TIA) after ion exchange chromatography (%CDT, Axis-Shield, Oslo, Norway); plasma γ-glutamyltransferase (GT) concentration was measured using enzymatic colorimetric assay, as standardised against IFCC (International Federation of Clinical Chemistry and Laboratory Medicine). More detailed information on GT and CDT measurements and on calculating the GT-CDT index has been previously reported^47^.

### The Finnish health-care system during the study

The production of public health care services was the municipalities’ responsibility during the study follow-up period^48^. Finland was divided into 21 hospital districts that provided specialised medical care for the population in their own areas, and approximately 160 health-care centres provided the primary health-care services described previously^22^. Employers were obligated to offer occupational health-care services for their employees in addition to the municipal system^48^. Financial incentive systems affecting what will be recorded were not in use in public or occupational health care. Primary health-care services could also be sought from private health care mainly financed by the patients’ own expence^48^. However, the availability of private health-care services during the study period was very limited in the study region compared to bigger cities. Consequently, most patients could use only public health-care services. Thus, in this and in our previous studies^22,23,32^, planned asthma follow-up contacts in health-care centres and in occupational health care were considered scheduled PHC contacts. All health-care centres in the region had respiratory nurses and a coordinator GP responsible for asthma management in the health-care centre, yet all GPs managed their own asthma patients during the study period. A common electronic patient record system was not yet used in the region, and professionals could use different and separate software in primary health-care centres, hospitals, and private health care. Our previous study also discussed the Finnish health-care system^22^.

### Statistical analysis

Continuous data are expressed as mean (SD) for variables with normal distribution and for parameters with skewed distributions medians, and 25–75 percentiles are shown. Group comparisons were performed by using Pearson Chi-square test for categorised variables. Two-sided *p-*values were used. A *P*-value < 0.05 was regarded as statistically significant. Statistical analyses were performed using SPSS software, version 27.0.1 (IBM SPSS, Armonk, NY).

### Reporting summary

Further information on research design is available in the Nature Research Reporting Summary linked to this article.

## Results

### Characteristics of the study population

Of the 203 total patients in SAAS study, 145 had scheduled office-based asthma contacts in PHC with a GP, nurse, or both. Most patients with PHC follow-up visits were female (63.4%). The mean age was 59.3 and BMI 28.4 at 12-year follow-up visit; thus, the study population was characterised with overweight. Half of the patients were ex- or current smokers, 37.4% were atopic (at least one positive skin prick test of common allergens), 69.7% had rhinitis, 8.3% had treated dyspepsia, and 31.0% of the patients had uncontrolled asthma according to GINA 2010^42^. The total adherence to ICS medication (ug budesonide equivalent dispensed/ug budesonide equivalent prescribed *100) during the 12 years was 81.3% among patients with scheduled office-based asthma contacts in PHC. Table 1 shows the characteristics of the study population at the 12-year follow-up visit. The Supplementary Material (eTable 2) shows the baseline characteristics of the 145 patients.

**Table 1:** Characteristics of the 145 patients with scheduled follow-up contacts in primary health care at 12-year follow-up visit.

	Patients (*n* = 145) with scheduled asthma follow-up contacts in primary health care
*Basic characteristics*	
Female *n (%)*	92 (63.4)
Age (y), *mean (SD)*	59.3 (13.2)
BMI (kg/m^2^), *mean (SD)*	28.4 (5.9)
Atopic *n (%*)^a^	49 (37.4)
Smokers (ex or current) *n (%)*	72 (49.7)
Alcohol heavy user *n (%)*	24 (16.6)
*Asthma severity n (%)*	
ACT score, *median (IQR)*	22 (19–24)
Uncontrolled asthma *n (%)*^b^	45 (31.0)
Severe asthma according to ERS/ATS criteria *n (%)*^*c*^	10 (6.9)
*Lung function & inflammation parameters*	
Pre-BD FEV_1_ (%), *mean (SD)*	87 (17)
Post-BD FEV_1_ (%), *mean (SD)*	90 (17)
Pre-BD FEV_1_/FVC, *median (IQR)*	0.73 (0.67–0.79)
Post-BD FEV_1_/FVC, *median (IQR)*	0.75 (0.69–0.80)
FeNO (ppb), *median (IQR)*	11 (5–19)
Blood eosinophils (×10^9^/l), *median (IQR)*	0.16 (0.10–0.27)
Total IgE (kU/l), *median (IQR)*	61 (23–153)
*Medication*	
Daily ICS in use *n (%)*	122 (84.1)
Daily SABA in use *n (%)*	21 (14.5)
Daily LABA in use *n (%)*	76 (52.4)
Daily add-on drug in use *n (%)*	82 (56.6)
Total adherence to ICS over 12 years, *median (IQR)*	81.3 (49.7–98.9)
≥1 oral corticosteroid course during 12-yr follow-up *n (%)*	49 (34.5)
*Comorbidities*	
Obesity (BMI ≥ 30 kg/m^2^) *n (%)*	47 (32.4)
Rhinitis *n (%)*	101 (69.7)
COPD *n (%)*	21 (14.6)
Diabetes *n (%)*	18 (12.4)
Hypertension *n (%)*	47 (32.4)
Ischemic heart disease *n (%)*	16 (11.0)
Any psychiatric disease *n (%)*	18 (12.4)
Treated dyspepsia *n (%)*	12 (8.3)
Number of comorbidities (COPD included), *median (IQR)*	1 (0–2)
*Health care use*	
Scheduled asthma contacts in PHC, *median (IQR)*	3 (1–6)
All-asthma-related health care contacts, *median (IQR)*	17 (11–24)
≥1 hospitalisation during 12-y *n (%)*	39 (26.9)

*BMI* body mass index, *ACT* asthma control test, *IQR* interquartile range, *BD* bronchodilator, *FEV*_*1*_ forced expiratory volume in 1 s, *FVC* forced vital capacity, *FeNO* fraction of nitric oxide in exhaled air, *ICS* inhaled corticosteroid, *SABA* short-acting β_2_-agonist, *LABA* long-acting β_2_-agonist. *Add-on drug* long-acting β_2_-agonist, leukotriene receptor antagonist, theophylline and/or tiotropium in daily use. *PHC* primary health care.

^a^At least one positive skin prick test of common allergens.

^b^Assessment of asthma control was performed according to the Global Initiative for Asthma (GINA) 2010 report.

^c^Assessment of severe asthma was performed according to the ERS/ATS severe asthma guideline 2014.

### Documentation of comorbidities and lifestyle factors in scheduled asthma contacts

All documented data was collected and analysed from the full 12-year follow-up period to evaluate the comorbidities and lifestyle factors assessments in scheduled asthma contacts in PHC. Documentation was seldom done for comorbidities such as obesity, overweight, rhinitis, sleep apnea, reflux symptoms, and intolerance to NSAIDs in the 542 scheduled asthma contacts in PHC. The occurrence of possible chronic or allergic rhinitis was documented in 8.9% of contacts and reflux symptoms in 1.1% of contacts (Table 2). Chronic or allergic rhinitis was mentioned in 35 subjects (24.1%) of the 145 patients with scheduled asthma contacts in PHC (eTable 3). Obesity or overweight were documented only in 0.9% to 1.3% of contacts, and the information on BMI was found in 1.5% of the contacts of the total 542 scheduled, office-based asthma contacts (Table 2). Recorded information on BMI was found in 8 patients (5.5%) out of 145 patients with scheduled asthma contacts in PHC. Overall, BMI and/or possible overweight or obesity were mentioned in 15 patients’ (10.3%) health records (eTable 3). Exercise habits were the most-often documented lifestyle factor, in 16.2% of the contacts (Table 2) and in 49 (33.8%) of the patients at least once (eTable 3). Dietary matters and alcohol consumption were rarely mentioned (Table 2).

**Table 2:** Documentation of comorbidities and lifestyle factors in scheduled asthma contacts (*n* = 542) and according to professional encountering the patient at follow-up contact.

	All scheduled PHC asthma contacts *n* = 542	GP contact *N* = 310	Nurse contact *N* = 103	Both GP and nurse *N* = 129	*P*-Value
*Comorbidity-related information recorded n (%)*					
BMI	8 (1.5)	3 (1.0)	3 (2.9)	2 (1.6)	0.365
Overweight	7 (1.3)	5 (1.6)	0	2 (1.6)	0.435
Obesity	5 (0.9)	4 (1.3)	0	1 (0.8)	0.485
Sleep apnea					
- suspected, not diagnosed	2 (0.4)	0	1 (1.0)	1 (0.8)	**0.019**
- diagnosed	4 (0.7)	0	3 (2.9)*	1 (0.8)	
Chronic/allergic rhinitis or its symptoms	48 (8.9)	30 (9.7)	6 (5.8)	12 (9.3)	0.481
Sinus infections or nasal polyps	29 (5.4)	19 (6.1)	3 (2.9)	7 (5.4)	0.450
Recurrent sinus infections	5 (0.9)	5 (1.6)	0	0	0.151
Reflux symptoms	6 (1.1)	5 (1.6)	1 (1.0)	0	0.335
NSAID intolerance	3 (0.6)	1 (0.3)	1 (1.0)	1 (0.8)	0.690
*Lifestyle-related factors recorded n (%)*					
Exercise habits	88 (16.2)	30 (9.7)	30 (29.1)*	28 (21.7)*	**<0.001**
Diet	5 (0.9)	1 (0.3)	1 (1.0)	3 (2.3)	0.135
Alcohol consumption	1 (0.2)	0	0	1 (0.8)	0.201

Statistically significant *p*-values are presented in bold.

*p < 0.05 compared to group doctor contacts.

We evaluated whether differences exist in the documentation of comorbidities or lifestyle factors according to who is responsible for the patient in the office-based asthma contacts; the GP, nurse, or both. However, no significant differences were found in recording comorbidities, but out of lifestyle factors, exercise habits were more-often mentioned (from 21.7% to 29.1%) if the nurse participated in the scheduled contact (Table 2).

### Documentation of asthma symptoms, medication, and patient guidance

**Fig. 2:**
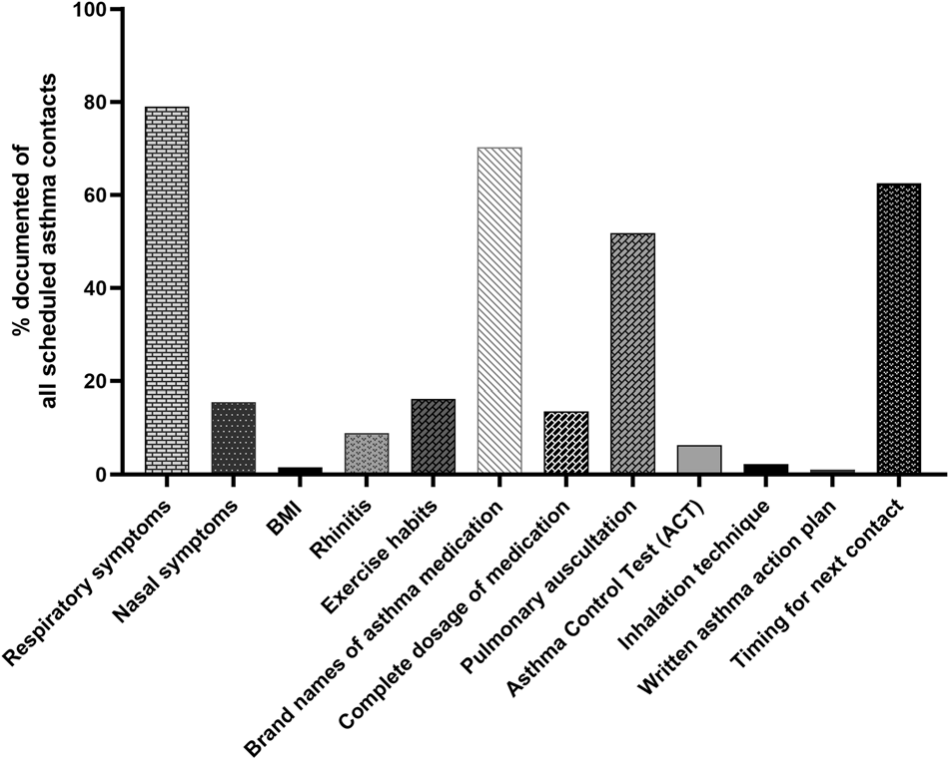
Documentation of asthma management details in scheduled asthma contacts in PHC during 12-year follow-up. The total number of contacts was 542.

Data on asthma management details (asthma symptoms, including ACT, medication, inhalation technique, patient guidance, etc.) during the follow-up period were collected and analysed. Figure 2 shows the documentation of collected asthma management details during scheduled asthma contacts (=542) in PHC. The occurrence of possible respiratory symptoms was recorded in 79.0% of visits and in 86.8% if both nurse and GP took part in the scheduled contact of the 542 scheduled PHC asthma contacts (Table 3). Nasal symptoms were mentioned in only 15.5% of the contacts (Table 3) and, overall, at least once in 52 patients (35.9%) (eTable 3). Data on the Asthma Control Test (ACT)^40^ was seldom found, in only 6.3% of contacts, but it was documented more often if both the nurse and GP participated in the contact (15.5%). Pulmonary auscultation data were registered in 72.9% of the physicians’ contacts.

**Table 3:** Documentation of asthma management details during scheduled asthma contacts (*n* = 542) and according to professional encountering the patient at follow-up contact.

	All scheduled contacts *N* = 542	GP contact *N* = 310	Nurse contact *N* = 103	Both GP and nurse *N* = 129	*P*-Value
*Asthma assessment performed*					
Respiratory symptoms *n* (%)	428 (79.0)	237 (76.5)	79 (76.7)	112 (86.8)*ǂ	**0.043**
Nasal symptoms *n* (%)	84 (15.5)	54 (17.4)	13 (12.6)	17 (13.2)	0.358
Work effect on symptoms assessed *n* (%)	53 (9.8)	37 (11.9)	5 (4.9)	11 (8.5)	0.096
Pulmonary auscultation *n* (%)	281 (51.8)	226 (72.9)ǂ∞	0	55 (42.6)ǂ	**<0.001**
ACT *n* (%)	34 (6.3)	5 (1.6)	9 (8.7)*	20 (15.5)*	**<0.001**
*Medication recorded*					
Asthma medication *n* (%)					
- Drug/brand names	381 (70.3)	198 (63.9)	77 (74.8)*	106 (82.2)*	**<0.001**
- Drug/brand names at least partially	483 (89.1)	273 (88.1)	88 (85.4)	122 (94.6)	0.056
- Complete dosage of asthma medication	73 (13.5)	49 (15.8)ǂ	8 (7.8)	16 (12.4)ǂ	**0.028**
- Dosage of asthma medication at least partially	350 (64.6)	207 (66.8)ǂ	54 (52.4)	89 (69.0)ǂ	**0.015**
- Inhalers	62 (11.4)	37 (11.9)	8 (7.8)	17 (13.2)	0.199
- At least some of the inhalers	183 (33.8)	114 (36.8)	25 (24.3)	44 (34.1)	0.067
Asthma medication changed in some way	145 (26.8)	112 (36.1)ǂ∞	5 (4.9)	28 (21.7)ǂ	**<0.001**
Possible side-effects evaluated	31 (5.7)	19 (6.1)	2 (1.9)	10 (7.8)	0.149
Inhalation technique *n* (%)					
- Mentioned	12 (2.2)	1 (0.3)	9 (8.7)*∞	2 (1.6)	**<0.001**
- Revised	12 (2.2)	1(0.3)	8 (7.8)*	3 (2.4)	<**0.001**
Nasal medication *n* (%)					
Started or changed	23 (4.2)	17 (5.5)ǂ	0	6 (4.7)	**0.024**
Already in use, no changes	75 (13.8)	34 (11.0)	16 (15.5)	25 (19.4)	
Medication for reflux symptoms *n* (%)					
Started	2 (0.4)	2 (0.6)	0	0	0.528
Already in use	7 (1.3)	5 (1.6)	0	2 (1.6)	
*Patient guidance recorded*					
Lifestyle factors *n* (%)					
- Recommendation to lose weight	5 (0.9)	4 (1.3)	0	1 (0.8)	0.485
- Recommendation to increase exercise	3 (0.6)	2 (0.6)	1(1.0)	0	0.579
- Recommendation to reduce alcohol use	1 (0.2)	0	1 (1.0)	0	0.118
Asthma action plan mentioned *n* (%)^a^	27 (5.0)	14 (4.5)	5 (4.9)	8 (6.2)	0.759
- Verbal AAP	22 (4.1)	14 (4.5)	3(2.9)	5 (3.9)	0.055
- Written AAP	2 (0.4)	0	0	2 (1.6)	
- Both verbal and written AAP	3 (0.6)	0	2 (1.9)	1 (0.8)	
Recommendation for the timing of the next scheduled contact *n* (%)	339 (62.5)	198 (63.9)	53 (51.5)	88 (68.2)ǂ	**0.025**

Statistically significant *p*-values are presented in bold.

*ACT* asthma control test, *AAP* asthma action plan.

*p < 0.05 compared to group doctor contacts.

^‡^*p* < 0.05 as compared to group nurse contacts.

^∞^*p* < 0.05 as compared to group both GP and nurse contact.

^a^Asthma action plan (AAP) = Written and/or verbally given description of how an individual should manage asthma, including advice for changes in medication, if necessary, and a plan for contact with the healthcare system.

The brand names of the entire asthma medication were recorded in 70.3% of all contacts (*n* = 542), while complete dosage of the medication and inhaler names or types were recorded less often in only 13.5% and 11.4% of all contacts. Overall, asthma medication data were mostly only partially documented and were more frequently mentioned if both professionals attended in the contact (Table 3). Changes in asthma medication were made in 26.8% of visits and more often during contacts when the GP was involved (36.1%). The information on inhalation technique revision was documented in only 2.2% of contacts (Table 3) and more by nurse (8.7%), but out of all 145 patients, it was revised in only 10 (6.9%) patients during 12-year follow-up (eTable 3). Regarding medication for comorbidities, medication for the nose was started or changed 23 times and twice for reflux symptoms in scheduled asthma contacts during the 12-year follow-up (Table 3). Nasal medication was documented at least once in 46 patients (31.7%) and reflux medication in 8 patients (5.5%) out of 145 patients (eTable 3).

Of all scheduled asthma contacts, the timing for the next scheduled follow-up contact was recommended in 62.5% of contacts and more often when the GP or both professionals were involved. In contrast, an asthma action plan (AAP) was recorded in only 5.0% of contacts (Table 3), and of all patients, only 24 (16.6%) had an AAP documented during the 12-year follow-up (eTable 3). Guidance on lifestyles (to lose weight, to increase exercise, or to reduce alcohol intake) was also rarely documented (Table 3).

## Discussion

In this 12-year, real-life, follow-up study we showed that comorbidities, lifestyle factors, inhalation technique, and asthma action plan were poorly documented during scheduled asthma contacts (*n* = 542) in PHC in Finland. The most frequently recorded asthma details were respiratory symptoms (79%), asthma medication brand names (70%), and the recommendation for the timing of the next follow-up contact (62.5%). All these details were found even more often if the nurse and GP both participated in the contact. Rhinitis was the most-often documented comorbidity, but it was registered only in 8.9% of all contacts. Recorded information on possible lifestyle guidance interventions given to the patients was found in <1% of contacts. Results from this longitudinal study may help to identify potential health-care practice-related causes of uncontrolled and difficult-to-treat asthma, and which areas require more urgent training and attention.

Obesity has been shown to be associated with uncontrolled and severe asthma^1–3,27,49–51^, poorer work ability^12^, lower lung function, more dispensed oral corticosteroids with higher doses, and higher health-care costs^50^, and it is a risk factor for asthma exacerbations even in patients with few symptoms^1^. Adult patients with asthma are at a higher risk of developing obesity^52^. Moreover, obesity has been shown to be a permanent problem in more than 85% of adult patients with asthma in long-term follow-up^50^. Weigh reduction in obese adults, also after bariatric surgery^53^, has shown to lead to overall improvement in asthma control, including airway hyper-responsiveness and inflammation^54^. We showed in this study that professionals rarely documented information about a patient’s BMI, overweight, or obesity. According to documented information, patients received no guidance in relation to obesity-related lifestyle factors during long-term follow-up, even though these factors are also shown to contribute to asthma independently. For example, low physical activity is associated with faster lung function decline^18^, dietary components are suggested to affect immune pathways in asthma^55^, and prolonged and heavy alcohol exposure may impair mucociliary clearance and may complicate asthma management^56^. A previous study based on physicians’ self-reports regarding clinical practice indicated that, overall, very few GPs assessed asthma patients’ lifestyle factors^34^, which is in line with our results. Overall, based on documented patient data, lifestyle factors were poorly registered; however, nurses mentioned exercise habits in almost every third contact. Lifestyle guidance was more the nurse’s responsibility in previous national and local asthma programmes, which may explain this result.

Allergic rhinitis is known as a predominant comorbid disease in difficult-to-treat asthma^36,49^. Chronic rhinosinusitis is known to be an independent predictor of asthma exacerbation among patients with difficult asthma^9,57^. Considering the unity of the upper and lower respiratory tract, the concept called ‘united airways’, screening and treating of rhinitis and other nasal conditions in asthma is important^57,58^. Thus, evaluating possible nasal symptoms and adherence to nasal medication should be assessed in every asthma contact. Medications treating nasal diseases have also been shown to be useful in improving control of asthma and reducing bronchial hyper-responsiveness^58^. A recent study showed that approximately 67% of the patients with moderate-severe rhinitis were not using the recommended intranasal corticosteroid therapy^36^. Aligning with previous studies^35,36^, our results showed that even though rhinitis is highly prevalent^49^, its screening and treatment in patients with asthma was suboptimal in PHC. In our study 70% of patients had rhinitis but it was recorded in less than every tenth and, overall, nasal symptoms less than in every fifth contact. The initiation of rhinitis treatment was rare. Based on recorded nasal medication data, over half of the patients with rhinitis may have been undertreated when medication for chronic rhinitis has been available only with a doctor’s prescription. Documentation of reflux symptoms, OSA and intolerance to NSAIDs was similarly underperformed, despite all these conditions being associated with severe asthma, poor symptom control, and more frequent exacerbations and hospitalisations^8,10,51,59,60^. NSAIDs (including aspirin) may exacerbate asthma symptoms in patients with N-ERD (NSAID-exacerbated respiratory disease), a chronic eosinophilic inflammatory disorder of the respiratory tract occurring in patients with asthma and/or rhinosinusitis with nasal polyps^10^. A recent study showed that the prevalence of N-ERD was 6.9% among asthmatics^60^, while the prevalence of gastroesophageal reflux varies between 17–74%^7,9^ and the prevalence of OSA ~39–50%^6,9^. Reflux disease and OSA may arguably have been underdiagnosed in our study population, considering a majority have a BMI > 25. OSA was probably not yet well known in PHC during the current study’s time period, and recognition improved after the national sleep apnea programme in Finland (2002–2010)^61^.

**Fig. 3:**
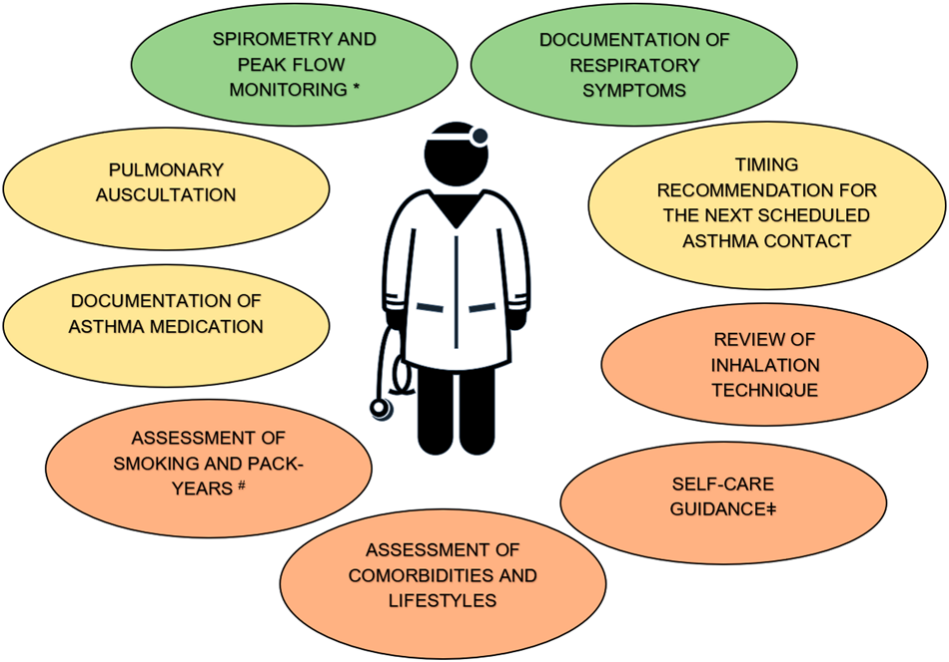
The content of the asthma follow-up contacts in PHC. Green colour describes the performed assessment that were implemented well, yellow colour describes moderate implementation, and the orange describes the measures that are poorly implemented. *Takala et al.^22^. ^#^Takala et al.^32^. ^ǂ^Self-care guidance includes patient asthma action plan instructions and lifestyle guidance.

The results in this and our previous studies^22,32^ suggest that implementation of the Finnish National Asthma Programme’s^31^ main objectives has been partially successful in PHC, but room still exists for improvement (Fig. 3). We found in this study that screening of asthma symptoms as a part of asthma control assessment has been managed well in PHC. Cloutier et al.’s previous study^30^ showed that physicians monitor selected symptoms depending on the symptom, from 48.4% to 56.0%. We were unable in this study to assess more precisely the extent of the symptoms’ evaluation and of the patients’ true symptom burden; thus, more research regarding this issue is needed in the future. Patients have been shown to overestimate their asthma control^36^, which supports assessing asthma control using objective methods such as lung function tests together with symptom questionnaires. ACT documentation was rarely found in our study, similar to previous studies in which validated patient-reported questionnaires were rarely used to monitor asthma control^28,30^. ACT was not yet in wide use in Finland during the SAAS study period, which probably explains our results to some extent. Pulmonary auscultation was recorded in almost 3 of 4 physicians’ contacts but never in nurses’ contacts, which is explained by the fact that pulmonary auscultation is usually performed only by a doctor in Finland.

It is essential that the complete asthma medication information, including names, doses and inhalers, is documented in patient records for continuity of care, because the professional responsible for patient care may change. The common electronic patient record system was not yet in use in our region during the SAAS study period, and some patients still had handwritten paper prescriptions in addition to those that were prescribed through the electronic patient health record system. As a result, the patient health record system did not necessarily have an up-to-date medication list or information about possible changes to medication made elsewhere, which also advocates for the importance of recording medication information. Asthma medication brand names were mentioned in 70% of scheduled contacts in our study, but dosage and inhalers were documented in only <14% of contacts. Only doctors had the right to prescribe medicines during the study period, which explains why medication changes were more common in visits when a GP was involved. This study and our previous studies^22,23^, show that patients with ≥2 scheduled contacts in PHC had high mean adherence to ICS medication (>80%), and their adherence level was higher compared to patients who had mainly follow-up contacts in secondary care (82% vs. 52%)^23^. Higher adherence was associated with non-controlled disease in SAAS-study population, while total adherence <80% was associated with more rapid lung function decline in not-controlled disease^62^. Our results suggest that professionals in PHC are good at promoting adherence to asthma medication. We were unable in this study, unfortunately, to assess in more detail how medication adherence was evaluated and if discussion supporting adherence to treatment, occurred at the contacts. The names of the medications in use were recorded well and adherence was high, so it can be assumed that treatment compliance in medication was discussed in the follow-up contacts to some extent. It could be speculated that continuity of care may be one reason for the good adherence when it was also shown that the recommendation for the timing of the next scheduled contact was documented in over 62% of contacts and in almost 70% if both professionals were attending.

Incorrect inhaler technique is common and can lead to poor asthma control^1^. Previous studies from Sweden and Finland showed that 87–97% of patients reported that they had received education about inhalation technique^24,63^. Another study from Australia revealed that patients overestimated the true success of their own inhalation technique when 73% of patients believed they did well, whereas an objective assessment showed that all patients had at least two errors and over 70% exhibited five or more errors^36^. In studies from the U.S. and Australia, 17–30% of PHC clinicians reported assessing inhaler technique^30,34^, but based on documented and reported patient data, only 1–5% of patients had their inhaler technique checked^21,36^, which is in line with our results. Checking the inhalation technique is usually the nurses’ task in the Finnish health care system, but still, according to recorded patient data, this was performed in approximately only 8% of nurse contacts, which is alarming.

AAP is a description of how an individual should manage asthma, including advice for medication changes, if necessary, and a plan for contact with the health-care system^20^. Use of written action plans is suggested to be poor both in PCH and in secondary care^33^ and shown to vary from 0 to 50%^21,28,30,33,34^. A previous study from Finland showed that over 78% of adult asthmatics reported having an asthma self-management plan^24^, but based on our results, AAP was not assessed or updated during planned contacts according to documented data. Recorded information on AAP was found in only 5% and written action plan in 1% of contacts, which can be considered surprising when one of the Finnish Asthma Programme’s most important goals was patients’ self-care guidance, including provision of both written and verbal asthma action plans^31^. Every patient in the SAAS study population received both verbal and literal asthma guidance, usually immediately upon asthma diagnosis confirmation in the respiratory department. Thus, could be argued whether the existence of an AAP was considered self-evident in PHC; however, it does not justify the omission of an AAP assessment. Chapman et al. suggested that physicians tend to rely upon advances in pharmacological intervention to improve the quality of asthma care rather than the non-pharmacological aspects of asthma management^28^. Our results showing that AAP and lifestyle interventions were poorly implemented in scheduled follow-ups in PHC support that. A recent UK study showed that many factors, such as poor attendance at asthma clinics, lack of time, demarcation of roles, limited access to a range of resources and competing agendas in consultations that are often due to multimorbidity, may increase the risk that self-care guidance is not provided during contacts^64^. These potential barriers are important to recognise when developing asthma monitoring and treatment guidance in the future.

This study’s major strength is its use of a real-life, unselected, adult-asthma population when patients with smoking or comorbidities were not excluded. Thus, our study population represents a typical PHC population with asthma^37,65^. Their asthma diagnosis was originally made by a respiratory physician based on typical symptoms and objective lung function measurements showing reversibility of airway obstruction^37^. All scheduled asthma contacts in PHC were evaluated in this study, including both nurse and GP visits, and the overall number of scheduled contacts may be expected to yield a representative sample of a real-life, adult-asthma population. We acknowledge that the significance of comorbidities in asthma control was perhaps not as well understood in 2002 compared to today. However, all the comorbidities with the exception of OSA, as well as other asthma management details evaluated in this study, have already been discussed in the first Finnish asthma guideline in 2000 and also e.g., in the GINA 2002 recommendation^66,67^. Therefore, it can be estimated that PHC has had opportunities to apply the best evidence-based practices during the study’s period. This study’s results are valuable because long-term, real-life, follow-up studies of adult-onset asthma in PHC are rare. Our results help to understand the possible health-care-related causes behind uncontrolled and difficult-to-treat asthma, e.g., which areas in assessing asthma require more specific training and attention.

A possible weakness of our study is that, e.g., comorbidities and other asthma-related details evaluated may have been screened and discussed during scheduled contacts or assessed earlier in other contexts, but these data have not been recorded. However, according to good clinical practice, the measures taken shall be recorded; otherwise, it can be interpreted that this has not been performed, or that the existence of the matter and its possible connection has not been considered. Additionally, regarding continuity of care, it is important that patient document entries are done well. We were unable in this study to assess more precisely either the extent of symptoms’ evaluation or the content of AAP instructions. Other important aspects of asthma care were not assessed in this study, such as exacerbations and trigger avoidance. More research is needed to evaluate these topics. Another limitation of our study is that our results may not represent Finland entirely, and it may not reflect the current situation, because the data were collected between 2002–2013. No common national asthma template is in use, and the recording practices may also differ regionally, e.g., due to different electronic health record systems. The use of ready-made phrase templates has become more common since the SAAS study period, which may have improved screening and assessment of asthma control-related issues. However, problems with accessibility to PHC have been increasing^48,68^, and it is very likely that asthma treatment and follow-up is largely carried out during visits for other conditions or for other reasons. A new, long-term follow-up study from the 2010s to 2020s would be needed to assess the current situation and whether asthma assessment has improved since the follow-up period in this study. Asthma control was defined according to GINA 2010 criteria at the 12-year follow-up visit, and asthma severity was classified according to the ERS/ATS 2014 guideline^42,43^. We consider it correct to use the data as they were collected and evaluated at the clinical visit on asthma control and as used in the original SAAS study material, even if asthma control and asthma severity criteria have change since then.

Regular monitoring is important when adult-onset asthma is often in non-remission^2–4^. The causes of poor asthma control can be complex^1,5^, and as shown in this study, based on documented patient data, the systematic assessment of asthma should be further improved in scheduled asthma contacts. However, our results also suggest that need exists to pay more attention to the quality of patient document entries in PHC in Finland^69^. Based on this study, the importance of screening and treating asthma-related comorbidities in PHC should be given more attention, especially those associated with uncontrolled and severe asthma. Documentation and follow-up of BMI data, together with guidance on healthy lifestyles and weight management, should be emphasised more in asthma guidelines as part of routine management. Reviewing asthma inhaler technique and patient self-care guidance are also central areas needing improvement. Based on these results, it is obvious that health-care personnel need continuous training in asthma management. In general, evaluation of lifestyle factors, patient guidance, lung function test performance, and revision of inhalation techniques have largely been the nurse’s responsibility, while the doctor’s task has been more to assess asthma control, medication, and patients’ personal treatment recommendation. The regular asthma follow-up could be carried out largely by the nurses, because not every patient needs a doctor’s assessment every year if their asthma is well controlled. Nevertheless, the nurse can gather information to assess asthma control and consult the doctor if needed. Asthma is one of our most common chronic diseases, but one could speculate whether its assessment is considered as important as, e.g., cardiovascular diseases, and whether possible multi-morbidities^11,14^ divert attention from asthma itself. The establishment of 21 well-being services counties to replace the former hospital districts since the beginning of 2023 in Finland has provided a new basis for developing uniform health-care services covering larger regions. It would be possible in this context to develop and update uniform asthma treatment chains covering entire regions and even to implement national asthma templates and educate professionals in systematic asthma assessment. This could improve asthma management. Further promoting the use of structured phrase templates could support asthma assessment in scheduled contacts, because it has been shown that evidence-based EMR interventions improve the asthma documentation and provision of asthma care^70^. In addition, shorter and clearly structured guidelines could be easier to implement in PHC^71^. Given the complexity of asthma care, sufficient time and resources for asthma assessment must be guaranteed for comprehensive evaluation and patient guidance to be successful. More research is needed to evaluate the overall asthma care that is currently obtained in all asthma-related contacts in PHC and to guide health-care personnel education regarding asthma monitoring in the future.

In conclusion, we showed in this real-life, 12-year, follow-up study that comorbidities, lifestyle factors, inhalation techniques, and asthma action plans were poorly documented in scheduled asthma contacts in PHC. Our results, based on documented patient data, suggest that the comprehensive assessment and guidance of asthma patients still needs to be improved in PHC.

### Supplementary information

Supplementary File

Reporting summary

## Data Availability

All data generated or analysed during this study are included in this published article and its Supplementary Information File. According to ethical permission and patient data-protection laws of Finland, single patient data cannot be made available.
