# Supplementary material for: Documentation of comorbidities, lifestyle factors, and asthma management during primary care scheduled asthma contacts
Source: NPJ Prim Care Respir Med. 2024 Mar 9;34:2. doi: 10.1038/s41533-024-00360-3 (PMC10925028; doi:10.1038/s41533-024-00360-3)
Supplement: Supplementary file 1 — Supplementary File [file 41533_2024_360_MOESM1_ESM.pdf]

Supplementary material

Documentation of comorbidities, lifestyle factors, and other asthma management details during scheduled asthma contacts in primary health care: 12-year follow-up study

Jaana Takala, MD, GP[1,2,3*], Iida Vähätalo, M.Sc.Pharm[1,3], Leena E. Tuomisto, MD, PhD[1,3], Onni Niemelä, MD, PhD[4,5], Pinja Ilmarinen, PhD[1,3], Hannu Kankaanranta, MD, PhD[1,3,6]

[1]Department of Respiratory Medicine, Seinäjoki Central Hospital, Wellbeing Services County of South Ostrobothnia, Seinäjoki, Finland

[2]Seinäjoki Health Care Centre, Wellbeing Services County of South Ostrobothnia, Seinäjoki, Finland

[3]Tampere University Respiratory Research Group, Faculty of Medicine and Health Technology, Tampere University, Tampere, Finland

[4]Department of Laboratory Medicine, Seinäjoki Central Hospital, Wellbeing Services County of South Ostrobothnia, Seinäjoki, Finland

[5]Tampere University, Tampere, Finland

[6]Krefting Research Center, Department of Internal Medicine and Clinical Nutrition, Institute of Medicine, Sahlgrenska Academy, University of Gothenburg, Gothenburg, Sweden

Corresponding author: Jaana Takala, MD, GP

Department of Respiratory Medicine

Seinäjoki Central Hospital

Hanneksenrinne 7

FIN-60220 Seinäjoki, FINLAND

e-mail: jaana.takala@fimnet.fi

Supplementary Table 1. Exclusion and inclusion criteria used in SAAS-study.

Inclusion criteria

- A diagnosis of new-onset asthma made by a respiratory specialist
- Diagnosis confirmed by at least one of the following objective lung function measurements
    - FEV_1 reversibility in spirometry of at least 15 % and 200 ml
    - Diurnal variability (⩾20%) or repeated reversibility (⩾15%/60 l/min) in PEF follow-up
    - A significant decrease in FEV_1 (15%) or PEF (20%) in response to exercise or allergen
    - A significant reversibility in FEV_1 (at least 15% and 200 ml) or significant mean PEF change in response to a trial with oral or inhaled glucocorticoids
- Symptoms of asthma
- Age ≥ 15 years

Exclusion criteria

- Physical or mental inability to provide signed informed consent
- Diagnosis of asthma below the age of 15 years

Reference: Kankaanranta, H., et al. Seinäjoki Adult Asthma Study (SAAS): a protocol for a 12-year real-life follow-up study of new-onset asthma diagnosed at adult age and treated in primary and specialised care. *NPJ Prim. Care Respir. Med.* **25:**15042 (2015).

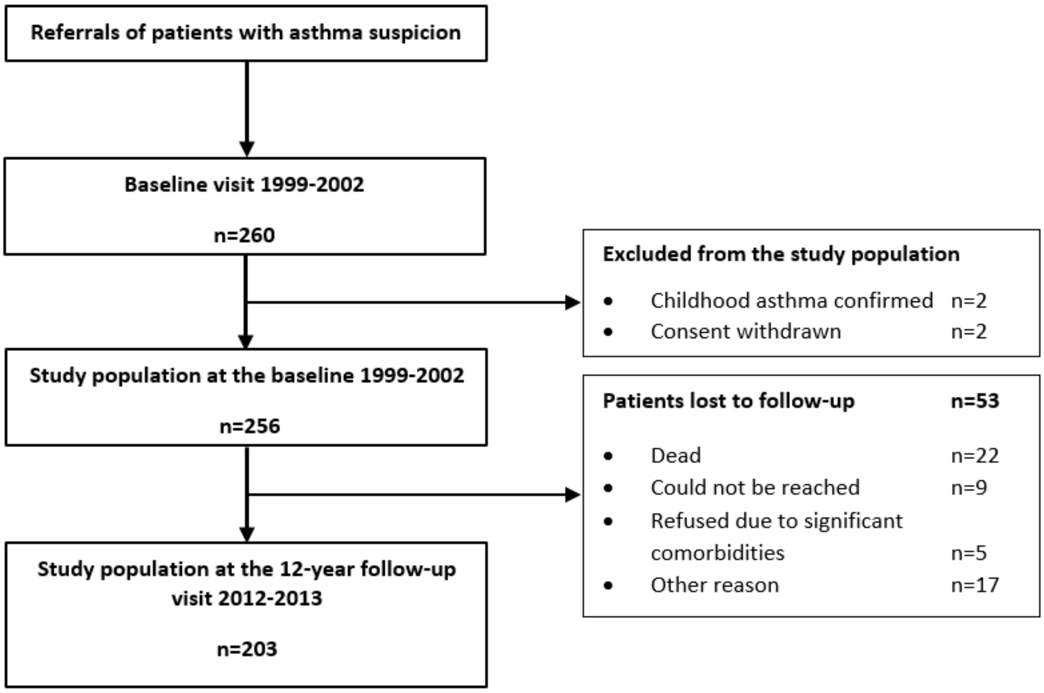

Supplementary Figure 1. Flowchart of Seinäjoki Adult Asthma Study.

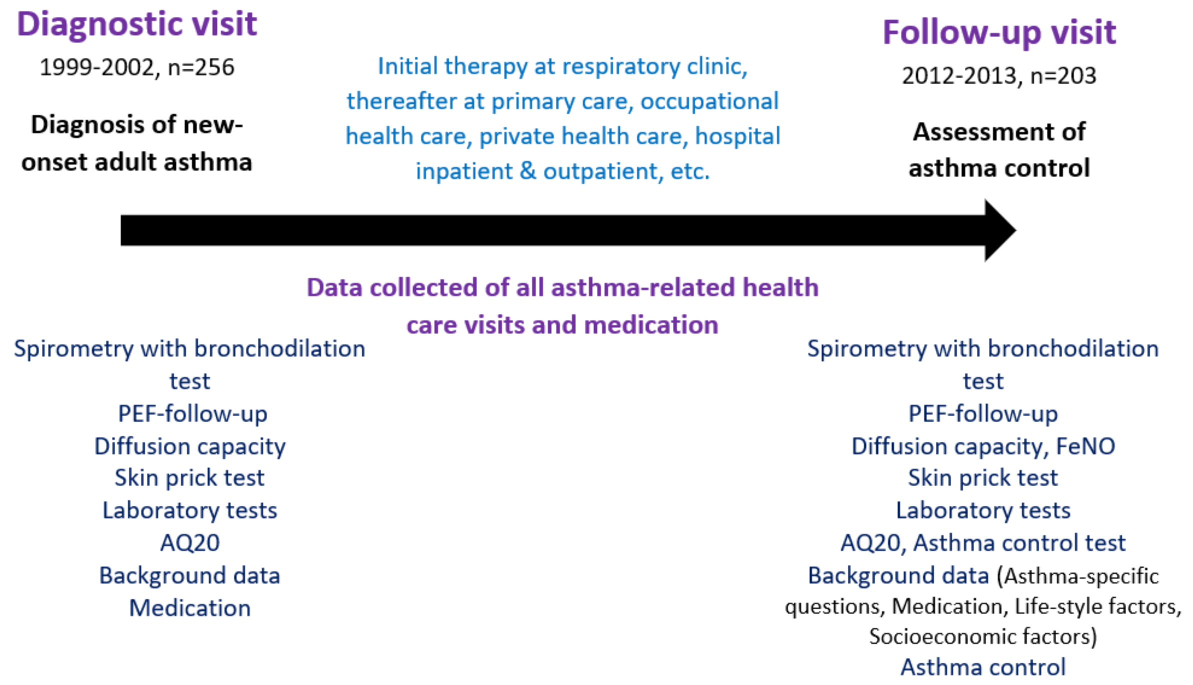

Supplementary Figure 2. Schematic presentation of Seinäjoki Adult Asthma Study.

Supplementary Table 2. Baseline characteristics of the 145 patients with scheduled follow-up contacts in primary health care.

	PATIENTS (n=145) WITH SCHEDULED ASTHMA FOLLOW-UP CONTACTS IN PRIMARY HEALTH CARE
**BASIC CHARACTERISTICS**	
Female *n (%)*	92 (63.4)
Age (y), *mean (sd)*	47.5 (13.1)
BMI (kg/m^2), *mean (sd)*	27.4 (5.1)
Atopic *n (%)* [a]	49 (37.4)
Smokers (ex or current) *n (%)*	69 (47.6)
**LUNG FUNCTION & INFLAMMATION PARAMETERS**	
Pre-BD FEV_1 (%), *mean (sd)*	81 (18)
Post-BD FEV_1 (%), *mean (sd)*	87 (18)
Pre-BD FEV_1/FVC, *median (IQR)*	0.75 (0.70-0.80)
Post-BD FEV_1/FVC, *median (IQR)*	0.79 (0.75-0.83)
Blood eosinophils (x10^9/l), *median (IQR)*	0.30 (0.15-0.44)
**COMORBIDITIES (self-reported data)**	
Obesity (BMI ≥ 30kg/m^2) *n (%)*	33 (22.8)
COPD *n (%)*	12 (8.4)
Diabetes *n (%)*	2 (1.4)
Hypertension *n (%)*	20 (13.8)
Ischemic heart disease *n (%)*	9 (6.2)

BMI = Body Mass Index, BD = bronchodilator, FEV_1 = forced expiratory volume in 1 s, FVC = forced vital capacity, [a] At least one positive skin prick test of common allergens.

Supplementary Table 3. Documentation of comorbidities and other asthma-related data among 145 patients with scheduled asthma contacts in PHC during 12-year follow-up.

	**PATIENTS (n=145) WITH SCHEDULED ASTHMA FOLLOW-UP CONTACTS IN PRIMARY HEALTH CARE**
**COMORBIDITY-RELATED INFORMATION n (%)**	
Obesity	4 (2.7)
Overweight	7 (4.8)
BMI	8 (5.5)
BMI and/or possible overweight/obesity	15 (10.3)
- BMI and overweight/obesity	3 (2.1)
Sleep apnea	
- suspected, not diagnosed	2 (1.4)
- diagnosed	4 (2.8)
Nasal symptoms	52 (35.9)
Chronic/allergic rhinitis or its symptoms	35 (24.1)
Sinus infections or nasal polyps	15 (10.3)
Recurrent sinus infections	3 (2.1)
Reflux symptoms	6 (4.1)
NSAID intolerance	3 (2.1)
**LIFESTYLE-RELATED FACTORS *n (%)***	
Exercise habits	49 (33.8)
- ≥2 times during follow-up	23 (15.9)
Diet	5 (3.4)
Alcohol consumption	1 (0.7)
**GUIDANCE WAS GIVEN *n (%)***	
to lose weight	5 (3.4)
to increase exercise	2 (1.4)
to reduce alcohol use	1 (0.7)
**INHALATION TECHNIQUE REVISED *n (%)***	10 (6.9)
**ASTHMA ACTION PLAN MENTIONED *n (%)***	24 (16.6)
**NASAL MEDICATION MENTIONED *n (%)***	46 (31.7)
**MEDICATION FOR GERD SYMPTOMS MENTIONED *n (%)***	8 (5.5)

BMI = Body Mass Index, NSAID=non-steroidal anti-inflammatory drug.
